# Comorbidity and Malnutrition as Key Determinants of Mortality in Elderly Haemodialysis Patients: A One‐Year Observational Study

**DOI:** 10.1002/jcsm.70062

**Published:** 2025-09-24

**Authors:** M. L. Sanchez‐Tocino, S. Mas‐Fontao, E. González‐Parra, P. Manso, M. Burgos, D. Carneiro, M. Pereira, C. Pereira, A. Lopez‐González, M. D. Arenas, on behalf the renal foundation work team

**Affiliations:** ^1^ Fundación Renal Madrid Spain; ^2^ Departamento de Enfermería y Fisioterapia Universidad de Salamanca Salamanca Spain; ^3^ Instituto de Investigación Sanitaria Fundación Jiménez Díaz (IIS‐FJD) Madrid Spain; ^4^ Centro de Investigación Biomédica en Red de Diabetes y Enfermedades Metabólicas Asociadas (CIBERDEM) Madrid Spain; ^5^ Facultad de Ciencias Biomédicas y de la Salud Universidad Alfonso X (UAX) Villanueva de la Cañada Spain; ^6^ Servicio de Nefrologia Fundación Jiménez Díaz Madrid Spain; ^7^ Hospital Universitario A Coruña A Coruña Spain

**Keywords:** Barthel, Charlson, elderly, Fried, haemodialysis, MIS, mortality

## Abstract

**Background:**

In elderly haemodialysis patients, poor functional status correlates with malnutrition, morbidity and mortality. This study aimed to assess 1‐year survival in an elderly haemodialysis population and evaluate the predictive capacity of commonly used scales for comorbidity, malnutrition, dependence and frailty.

**Methods:**

We conducted a 1‐year observational study (2022) on prevalent haemodialysis patients aged > 75 years with > 3 months of treatment. Mortality was assessed during the follow‐up. We analysed sociodemographic, dialysis‐related, analytical and lifestyle variables. Additionally, we evaluated comorbidity (Charlson), dependence (Barthel), malnutrition‐inflammation (MIS scale) and frailty (FRIED). Statistical analysis included comparisons between continuous variables using the Mann–Whitney *U* test or Student's *t* test, based on normality distribution. Categorical variables were compared using the chi‐square test. Kaplan–Meier survival analysis with log‐rank test was used to compare survival curves. Multivariate analysis was performed using Cox proportional hazards models, adjusting for confounders including dialysis adequacy and underlying kidney disease aetiology. Optimal cutoff points for predicting mortality were determined using receiver operating characteristic (ROC) curves.

**Results:**

A total of 107 haemodialysis patients (57% male, mean age 81.3 ± 4.53 years, mean time on haemodialysis 51.71 ± 51.04 months) were included. Sixteen patients (15%) died within 1 year. Deceased patients were older (83.94 ± 4.52 vs. 80.34 ± 4.39 years, *p* = 0.011) and had longer dialysis duration (76.46 ± 54.73 vs. 47.35 ± 49.4 months, *p* = 0.035), lower albumin (3.51 ± 0.54 vs. 3.86 ± 0.31 g/dL, *p* < 0.001) and lower creatinine levels (5.48 ± 1.12 vs. 6.50 ± 1.67 mg/dL, *p* = 0.021). They scored higher on all four scales analysed: Charlson (10.94 ± 1.81 vs. 8.95 ± 1.92, *p* < 0.001), MIS (11.31 ± 4.22 vs. 6.07 ± 3.27, *p* < 0.001), Barthel (52.50 ± 27.2 vs. 78.41 ± 23.11, *p* < 0.001), and FRIED (3.19 ± 1.05 vs. 2.18 ± 1.32, *p* = 0.005). Institutionalization (*p* = 0.004), inability to walk (*p* < 0.001) and stretcher transport (*p* < 0.001) were also significantly associated with mortality. The optimal cutoff points for predicting mortality were Charlson index ≥ 9.5 (AUC 0.788, 95% CI: 0.65–0.88), MIS ≥ 7.5 (AUC 0.844, 95% CI: 0.73–0.93), Barthel ≤ 67.5 (AUC 0.79, 95% CI: 0.68–0.79) and FRIED ≥ 2.5 (AUC 0.719, 95% CI: 0.56–0.83). In multivariable analysis, Charlson ≥ 9.5 (HR 2.75, 95% CI: 0.83–9.06, *p* = 0.096) and MIS ≥ 7.5 (HR 8.15, 95% CI: 1.10–60.58, *p* = 0.040) remained significant predictors of mortality.

**Conclusions:**

The Charlson, Barthel, MIS and FRIED scales are useful tools for predicting mortality in elderly haemodialysis patients, with defined cutoff points for increased mortality risk. We have defined specific cutoff points that determine increased mortality risk in this population. However, comorbidity and malnutrition showed the strongest independent predictive value. These findings highlight the need for targeted interventions to address malnutrition and comorbidity in this vulnerable population.

## 1 Introduction

As life expectancy increases and lifestyle‐related diseases become more prevalent, the incidence of chronic kidney disease (CKD) is rising [1]. The ageing of the general population is evident in haemodialysis (HD) units. Continuous advances in dialysis techniques have contributed to improved patient outcomes. Additionally, improvements in anaemia correction, secondary hyperparathyroidism management and the implementation of prevention programmes in healthcare systems have further enhanced survival rates [2, 3, 4, 5].

An individual's functional status reflects their ability to perform activities that meet basic daily living needs, continue life roles and maintain health and well‐being. Poor functional status is associated with lower survival in older adults and patients with chronic diseases, determining quality of life. As with other chronic diseases, low functionality correlates with morbidity and mortality in elderly CKD patients [6, 7, 8].

It is challenging to establish how to measure functionality in elderly HD patients. There are numerous scales that assess functional decline, activity limitations and their impact on quality of life as well as morbidity and mortality. These scales are essential for assessing patient functionality: the Charlson Comorbidity Index measures the burden of comorbidities; the MIS provides a quantitative evaluation of nutritional status and inflammation; the Barthel Index assesses independence in daily activities; and the FRIED scale identifies frailty based on physical performance.

The Charlson Comorbidity Index evaluates the burden of comorbidities and their impact on mortality, though its applicability in elderly populations has limitations due to underestimation of prevalent conditions such as dementia, anaemia or depression [9]. The Malnutrition‐Inflammation Score (MIS) assesses nutritional status and inflammation through clinical and biochemical parameters, both of which are critical in dialysis patients due to their association with morbidity and survival [10]. The Barthel Index measures independence in basic activities of daily living, offering insight into the patient's functional capacity and rehabilitation potential [11]. Lastly, the FRIED frailty scale identifies frailty based on physical performance criteria, highlighting individuals at higher risk of adverse health outcomes [12]. These tools provide a comprehensive approach to evaluating functionality in this vulnerable population.

These scales present different cutoff points that determine degrees of dysfunction. Their relationship with mortality has been studied, including which best determine the risk of death associated with dysfunction [13, 14].

However, the cutoff points established for these scales were designed to measure the general population of all ages and those without kidney disease. HD patients have special characteristics that may determine mortality differently from individuals without kidney disease.

Given the high mortality risk associated with dialysis‐dependent CKD, a 1‐year observational study offers a relevant timeframe to assess survival outcomes. Haemodialysis patients exhibit substantially higher mortality rates than the general population [15], making a 1‐year period both practical and clinically meaningful for capturing prognostic data. Additionally, a 1‐year period aligns with standard clinical assessments, allowing for a practical and comprehensive evaluation of functionality and its impact on survival. This study advances current research by defining specific cutoff points for common assessment scales, thereby refining risk stratification and guiding targeted interventions in elderly HD patients.

The objective of this study was to describe the characteristics of the elderly population on haemodialysis and study their 1‐year survival. We evaluated the ability to predict mortality using common scales for comorbidity, malnutrition, dependence and frailty routinely used in haemodialysis units. By doing so, this study provides new insights into the applicability and prognostic relevance of these assessments, contributing to the optimization of risk stratification in clinical practice.

## 2 Methods

### 2.1 Patients Study Design and Population

This observational descriptive study was conducted over 1 year (January to December 2022) on patients from the chronic haemodialysis programme of four outpatient centres and one hospital unit of the Spanish Renal Foundation. From the total patients undergoing dialysis in these units, those aged over 75 years, who had been on the programme for more than 3 months, and who had accepted and signed informed consent were included in the study. Patients with cognitive impairment that prevented them from providing informed consent, as well as those who declined participation, were excluded.

### 2.2 Variables and Measurements Instruments

The study focused on four main variables: comorbidity, malnutrition‐inflammation, dependence and frailty. These variables were measured at the beginning of the study. Subsequent measurements were not performed; only baseline data and mortality at 12 months were recorded. Mortality data were obtained through the Nefrosoft electronic medical record system.

The scales used for each variable and their cutoff points are described below:

- *Comorbidity*: Assessed using the Charlson Comorbidity Index, a tool designed to classify comorbidities based on their severity and impact on mortality risk. Although it was originally developed to predict 10‐year survival, its utility in elderly populations has been questioned due to its underestimation of prevalent conditions such as dementia, anaemia and depression. For dialysis patients, various cutoff points have been reported for HD populations; our selected threshold is supported by previous literature. Comorbidity scores above 6 indicate high risk [16], with significant increases in 1‐year mortality observed in those scoring above 8 [17].
- *Malnutrition‐inflammation*: Evaluated using the Malnutrition‐Inflammation Score (MIS), which is a validated quantitative measure derived from a subjective global assessment. The MIS includes 10 components, such as weight change, appetite, gastrointestinal symptoms and various biochemical markers, yielding a total score ranging from 0 to 30. Scores above 10 indicate severe malnutrition, whereas scores below 2 suggest normal nutrition [10, 18].
- *Dependence*: Measured by the Barthel Index, which assesses the ability to perform basic activities of daily living (ADLs). The index scores range from 0 to 100, with lower scores indicating higher levels of dependence. Scores below 20 suggest total dependence, whereas a score of 100 indicates full independence. This index is crucial for understanding the patient's functional status and potential for rehabilitation [11, 19].
- *Frailty*: Assessed using the FRIED scale, which defines frailty as the presence of three or more of the following criteria: unintentional weight loss (> 4.5 kg or > 5% of body weight in the last year), self‐reported exhaustion (evaluated through two questions from the Center for Epidemiological Studies‐Depression scale), weakness (grip strength), slow walking speed and low physical activity. Grip strength was measured using a dynamometer, taking the highest value from three attempts in each hand, adjusted for sex and BMI. Slow walking speed was assessed by measuring the time to walk 4.6 m at a usual pace, with thresholds adjusted for sex and height. Low physical activity was estimated based on weekly energy expenditure, with specific cutoffs for men and women. These criteria follow the original validation from the Cardiovascular Health Study and have been widely used in dialysis populations [12].

### 2.3 Additional Variables

Other collected variables included demographic data, clinical characteristics, dialysis parameters, anthropometric measurements and relevant laboratory values, such as albumin, total iron binding capacity (TIBC) and creatinine levels. Dialytic efficacy was measured using the Daurgidas Kt/V formula.

### 2.4 Data Collection and Analysis

Demographic and clinical data were obtained from medical records. For survival analysis, the population was grouped into categories based on the severity of their comorbidity, malnutrition, dependence and frailty. The median Charlson score was used as a cutoff point due to the influence of age on comorbidity. Dialysis‐related variables included the duration of haemodialysis sessions, weekly hours and frequency. Additional factors such as institutionalization, mode of transport to dialysis and posttreatment fatigue were also documented.

This comprehensive approach allowed for a detailed assessment of the patients' health status and its impact on mortality.

### 2.5 Statistical Analysis

Chi‐square test was used for categorical variables, Student's *t* test (parametric) or Mann–Whitney *U* test (nonparametric) for continuous variables and one‐way ANOVA for multiple comparisons. Normality of distribution was assessed using the Kolmogorov–Smirnov test. Survival analysis was performed using Kaplan–Meier survival curves and log‐rank test. Multivariate analyses were conducted using Cox proportional hazards models, adjusting for potential confounders such as dialysis adequacy (number of session and KTV) and underlying causes of kidney failure. Analysis of potential interactions between the scales did not yield statistically significant results, suggesting that the compounded effects of high comorbidity and frailty may not be synergistic in our cohort.

Quantitative variables were presented as mean and standard deviation. Qualitative variables were presented as absolute numbers and percentages. Statistical significance was set at *p* < 0.05. Statistical analysis was performed using IBM SPSS Statistics V20.

## 3 Results

### 3.1 Study Population

A sample size of 107 patients was considered sufficient based on preliminary data [20, 21] and previous literature [22, 23, 24], ensuring adequate power to detect significant differences in mortality risk. In this population, 57% were men; the mean age was 81.3 ± 4.53 years, and the mean time on HD was 51.71 ± 51.04 months. The aetiologies of kidney disease were diabetes mellitus 24 (22.4%), unknown kidney disease 25 (23.4%), vascular 25 (23.4%), tubulointerstitial nephritis 7 (6.5%), glomerular 12 (11.2%), polycystic kidney disease 8 (7.5%) and others 6 (5.6%).

Table 1 shows the characteristics of the study population in terms of demographic data, kidney disease and HD regimen, anthropometric data, laboratory values and scores on the measurement scales used. Differences by sex were studied. Men dialyzed for longer periods related to their larger body surface area and had higher blood creatinine values. Regarding scores on the measured scales, women presented lower comorbidity. There were no differences in total scores for the other scales.

**TABLE 1:** Demographic data, kidney disease and haemodialysis regimen, anthropometric measurements, lab results and scores on comorbidity, malnutrition‐inflammation, dependency and frailty scales. Mean ± SD.

	Total *n* = 107 (100%)	Men *n* = 61 (57%)	Women *n* = 46 (43%)	*p*
Demographic data				
Age (years)	81.3 ± 4.535	80.9 ± 4.2	81.8 ± 4.87	0.318
Time on dialysis (months)	51.71 ± 51.04	43.7 ± 39.6	62.3 ± 61.1	0.062
Haemodialysis regimen and residual diuresis				
Time on HD per session (hours)	3.6 ± 0.46	3.8 ± 0.45	3.5 ± 0.47	0.058
Time on HD ≥ 4 h (yes)	50/107 (47%)	34/61 (56%)	16/46 (35%)	0.031
Time on HD per week (hours)	11 ± 1.74	11.2 ± 1.9	10.8 ± 1.5	0.292
Time on HD per week > 12 h (YES)	49/107 (46%)	33/61 (54%)	16/46 (35%)	0.047
Diuresis > 500 mL/day	68/107 (64%)	41/61 (67%)	27/46 (59%)	0.365
Anthropometry				
Albumin (g/dL)	161.97 ± 8.84	167 ± 6.92	155.35 ± 6.75	< 0.001
Weight (kg)	68.02 ± 12.07	72.31 ± 10.03	62.34 ± 11.35	< 0.001
Body mass index (kg/m^2^)	25.95 ± 4.28	25.93 ± 3.66	25.92 ± 5.02	0.959
Lab results				
Albumina (g/dl)	3.81 ± 0.37	3.84 ± 0.35	3.76 ± 0.41	0.265
Albumin > 3.5 mg/dL	82/107 (76.6%)	48/61 (79%)	34/46 (74%)	0.563
Creatinine (mg/dl)	6.35 ± 1.63	6.67 ± 1.68	5.92 ± 1.47	0.018
KTV > 1.3	83/107 (85.6%)	46/61 (82%)	37/49 (90%)	0.262
Scales assessment				
Charlson comorbidity (pts.)	9.24 ± 2.02	9.64 ± 2.21	8.72 ± 1.63	0.019
MIS nutrition (pts.)	6.85 ± 3.89	6.34 ± 3.61	7.52 ± 4.18	0.121
Barthel dependency (pts.)	74.53 ± 25.38	76.72 ± 25.51	71.63 ± 25.19	0.307
Fried frailty (pts.)	2.33 ± 1.33	2.13 ± 1.23	2.59 ± 1.42	0.079

*Note*: Scale classifications: Charlson: low comorbidity (< 9 pts.), high comorbidity (> 9 pts.); MIS: extremely malnourished (> 10 pts.); very severe malnutrition (7–10 pts.); moderate to severe malnutrition (5–7 pts.); mild to moderate malnutrition (2–5 pts.); well‐nourished (< 2 pts.); Barthel: independent (100 pts.); mild dependency (91–99 pts.); moderate dependency (61–90 pts.); severe dependency (21–60 pts.); total dependency (< 20 pts.); Fried: not frail (0 pts.); prefrail (1–2 pts.); frail (> 3 pts.); Statistical significance: *p* < 0.05, men vs. women.

Abbreviation: TIBC, total iron‐binding capacity.

Regarding other recorded variables, 40 (67%) were dialyzed through a catheter. Sixty eight (64%) of the patients urinated. A total of 38 patients (36%) exhibited a residual urine output exceeding 500 mL per day. Of the total patients, 87 (82%) could walk, and 87 (82%) were transported to the centre by ambulance, the rest by their own means. Only nine (4%) were transported reclined on a stretcher due to their dependent state. Nine (8%) of the patients lived in nursing homes, and 60 (56%) reported extreme fatigue after dialysis treatment.

Finally, at 12 months from data collection, 16 (15%) of the patients had died.

### 3.2 Comorbidity, Malnutrition, Dependence, and Frailty Scales

Table 2 classifies the population according to the cutoff points established by the scales themselves. No differences were found between sexes.

**TABLE 2:** Percentages by severity degrees of comorbidity, malnutrition, dependency and frailty scales.

Classification		Total *n* = 107 (100%)	Normal‐mild/moderate–severe	Men *n* = 61 (57%)	Women *n* = 46 (43%)	*p*
Charlson comorbidity	Low comorbidity	44/107 (41%)	44/107 (41%)	21/44 (48%)	23/44 (52%)	0.105
	High comorbidity	63/107 (59%)	63/107 (59%)	40/63 (64%)	23/63 (36%)	
MIS malnutrition	Well‐nourished	11/107 (10%)	48/107 (45%)	30/48 (63%)	18/48 (37%)	0.301
	Mild–moderate malnutrition	37/107 (35%)				
	Moderate–severe malnutrition	18/107 (17%)	59/107 (55%)	31/59 (53%)	28/59 (47%)	
	Very severe malnutrition	19/107 (18%)				
	Extremely malnourished	22/107 (21%)				
Barthel dependency	Independent	27/107 (25%)	85/107 (79%)	50/85 (59%)	35/85 (41%)	0.478
	Mild dependency	58/107 (54%)				
	Moderate dependency	9/107 (8%)	22/107 (21%)	11/22 (50%)	11/22 (50%)	
	Severe dependency	7/107 (6%)				
	Total dependency	6/107 (5%)				
Frail_ fragilidad	Not frail	9/107 (8%)	58/107 (54%)	38/58 (66%)	20/58 (34%)	0.053
	Pre‐frail	49/107 (46%)				
	Frail	49/107 (46%)	49/107 (46%)	23/49 (47%)	26/49 (53%)	

*Note*: Classification scales: CHARLSON: low comorbidity (< 8 pts.), high comorbidity (> 8 pts); MIS: extremely malnourished (> 10 pts.), very severe malnutrition (7–10 pts.), moderate–severe malnutrition (5–7 pts.), mild–moderate malnutrition (2–5 pts.), well‐nourished (< 2 pts.); Barthel: independent (100 pts.), mild dependency (91–99 pts.), moderate dependency (61–90 pts.), severe dependency (21–60 pts.), total dependency (< 20 pts.); Fried: not frail (0 pts.), prefrail (1–2 pts.), frail (> 3 pts.). Statistical significance: *p* < 0.05, men vs. women.

### 3.3 Mortality Analysis

Table 3 presents the association between mortality and demographic data, haemodialysis regimen, anthropometric data, laboratory values and scores on the comorbidity, malnutrition‐inflammation, dependence and frailty assessment scales. The deceased were older, had been on dialysis longer and had lower albumin and creatinine levels than survivors. Significant differences were also observed in all measured scales, indicating higher mortality rates in patients with elevated comorbidity, greater malnutrition, increased dependence and higher frailty scores.

**TABLE 3:** Relationship between mortality and demographic data, haemodialysis regimen, anthropometrics, analytical data and comorbidity, malnutrition‐inflammation, dependency and frailty assessment scores. Mean ± SD.

	Total *n* = 107 (100%)	Deceased *n* = 16 (15%)	Alive *n* = 91 (85%)	*p*
Demographic data				
Age (years)	81.3 ± 4.53	83.94 ± 4.52	80.34 ± 4.39	0.011
Sex = male (%)	61	8/61 (13%)	53/61 (87%)	0.591
Haemodialysis regimen and residual diuresis				
Time on dialysis (months)	51.71 ± 51.04	76.46 ± 54.73	47.35 ± 49.4	0.035
Time on HD per session (hours)	3.6 ± 0.46	3.6 ± 0.50	3.59 ± 0.46	0.934
Time on HD per week (hours)	11 ± 1.74	11.28 ± 0.94	10.96 ± 1.85	0.506
Residual diuresis = yes (%)	68	9/68 (13%)	59/68 (87%)	0.578
Anthropometrics				
Height (cm)	161.97 ± 8.84	159.94 ± 8.26	161 233 ± 9.05	0.326
Weight (kg)	68.02 ± 12.07	66.5 ± 14.23	68.29 ± 11.72	0.587
Body mass index (kg/m^2^)	25.95 ± 4.28	25.83 ± 421	25.99 ± 4.31	0.911
Analytical data				
Albumin (g/dl)	3.81 ± 0.37	3.51 ± 0.54	3.86 ± 0.31	< 0.001
TIBC (mcg/dl)	249.01 ± 99.53	247.19 ± 74.93	249.42 ± 103.58	0.934
Creatinine (mg/dl)	6.35 ± 1.63	5.48 ± 1.12	6.50 ± 1.67	0.021
*Kt*/*V*_*urea*_	1.65 ± 0.35	1.74 ± 0.49	1.62 ± 0.32	0.243
Assessment scales				
Charlson comorbidity (pts.)	9.24 ± 2.02	10.94 ± 1.81	8.95 ± 1.92	< 0.001
Charlson < 9	44	2/44 (4%)	44/44 (96%)	0.013
Charlson ≥ 9	63	14/63 (22%)	49/63 (78%)	
MIS nutrition (pts.)	6.85 ± 3.89	11.31 ± 4.22	6.07 ± 3.27	< 0.001
Normonutritional	11	0/11 (0%)	11/11 (100%)	< 0.001
Mild malnutrition	37	2/37 (5%)	35/37 (95%)	
Moderate malnutrition	18	0/18 (0%)	18/18 (100%)	
Severe malnutrition	19	4/19 (21%)	15/19 (79%)	
Extreme malnutrition	22	10/22 (46%)	12/22 (54%)	
Barthel dependency (pts.)	74.53 ± 25.38	52.50 ± 27.2	78.41 ± 23.11	< 0.001
Independent	27	0/27 (0%)	27/27 (100%)	0.004
Mild dependency	58	8/58 (14%)	50/58 (86%)	
Moderate dependency	9	2/9 (22%)	7/9 (78%)	
Severe dependency	7	3/7 (43%)	4/7 (57%)	
Total dependency	6	3/6 (50%)	3/6 (50%)	
Fried frailty total (pts.)	2.33 ± 1.33	3.19 ± 1.05	2.18 ± 1.32	0.005
Nonfrail	9	0/9 (0%)	9/9 (100%)	0.032
Prefrail	49	4/49 (8%)	45/49 (92%)	
Frail	49	12/49 (25%)	37/49 (75%)	
Lifestyle				
Ability to walk				
Yes	87	7/87 (9%)	80/87 (92%)	< 0.001
No	20	9/20 (45%)	11/20 (55%)	
Lives in a care home				
Yes	9	5/9 (56%)	4/9 (44%)	0.004
No	98	11/98 (11%)	87/98 (89%)	
Transport to HD centre and home				
Ambulance lying down	3	3/3 (100%)	0/0 (0%)	< 0.001
Ambulance sitting	82	11/82 (13%)	71/82 (87%)	
Own means	22	2/22 (9%)	20/22 (91%)	
Extreme fatigue posttreatment				
Yes	60	11/60 (18%)	49/60 (82%)	0.292
No	47	5/47 (11%)	42/47 (89%)	

*Note*: Scale classifications: Charlson: low comorbidity (< 9 pts.), high comorbidity (> 9 pts.); MIS: extremely malnourished (> 10 pts.); very severe malnutrition (7–10 pts.); moderate to severe malnutrition (5–7 pts.); mild to moderate malnutrition (2–5 pts.); well‐nourished (< 2 pts.); Barthel: independent (100 pts.); mild dependency (91–99 pts.); moderate dependency (61–90 pts.); severe dependency (21–60 pts.); total dependency (< 20 pts.); Fried: not frail (0 pts.); prefrail (1–2 pts.); frail (> 3 pts.). Statistical significance: *p* < 0.05, men vs. women.

Abbreviation: TIBC, total iron‐binding capacity.

Inability to walk, transport by ambulance in a reclined position and living in a nursing home were associated with higher mortality. However, postdialysis fatigue did not show significant differences between those who died and those who survived at 1 year of follow‐up.

**FIGURE 1:**
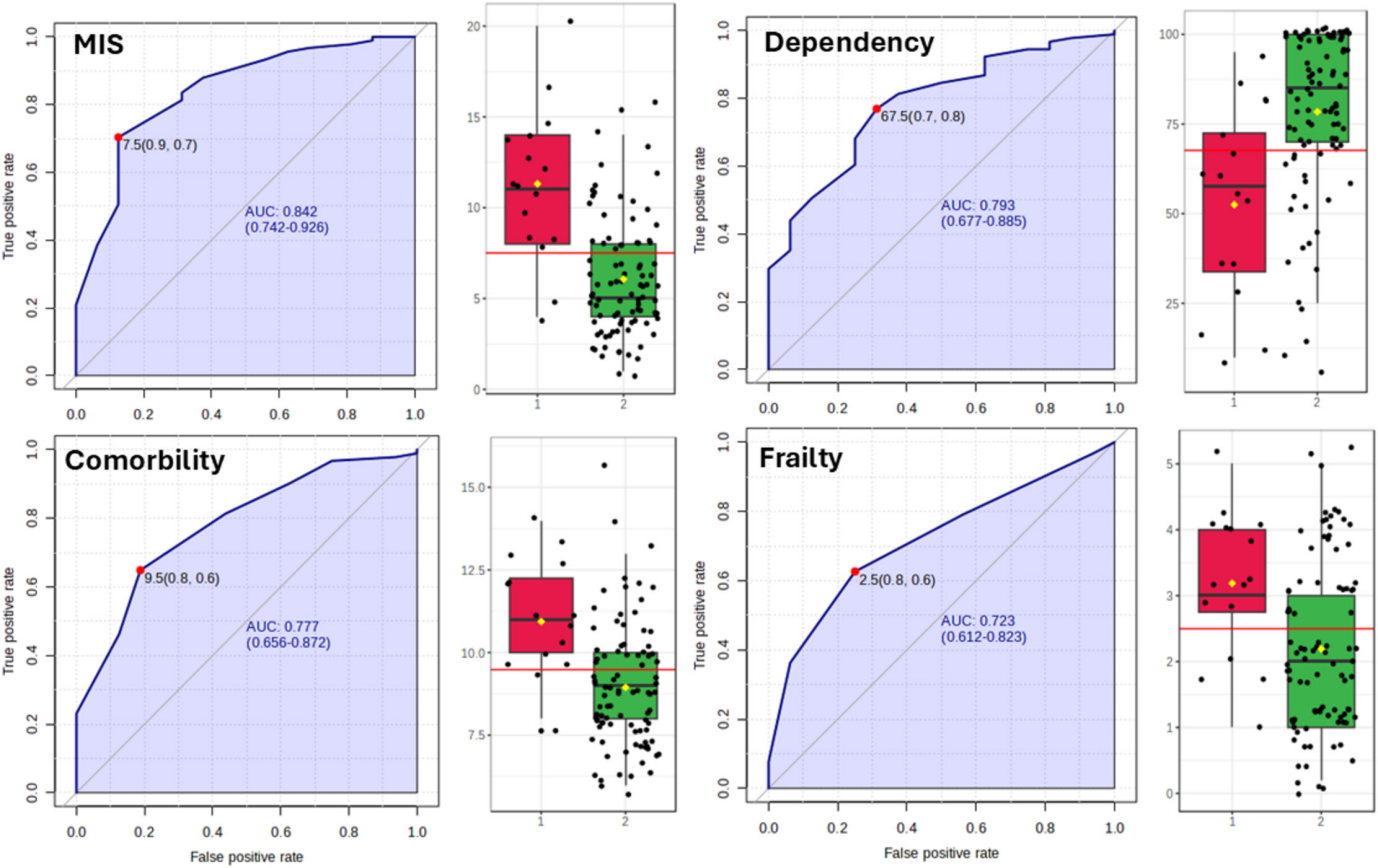
ROC curves and boxplots for mortality predictive scales in elderly haemodialysis patients.

Figure 1 represents the ROC curves that identify the cutoff point determining mortality for each of the scales studied in our population.

**FIGURE 2:**
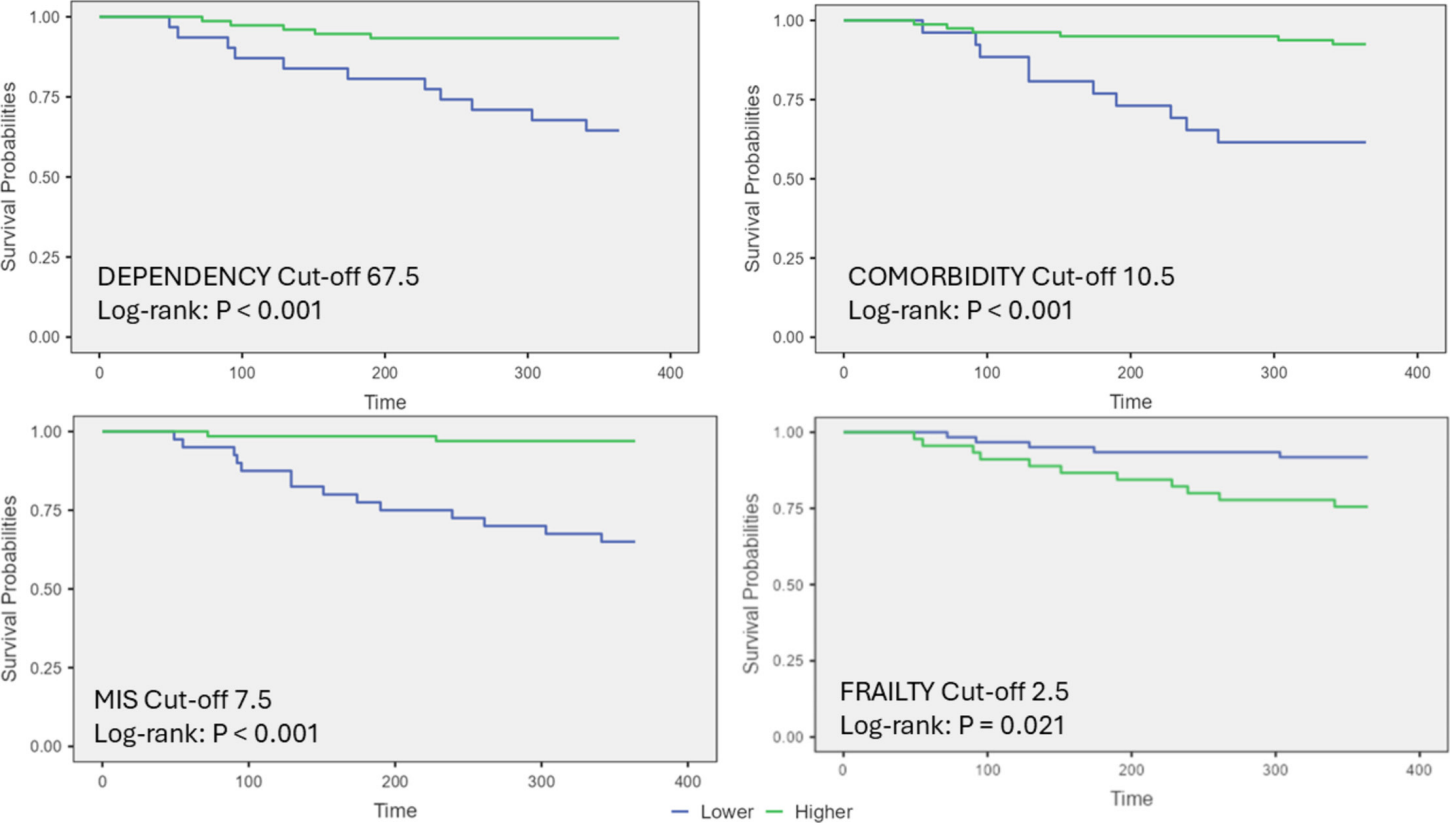
Kaplan–Meier survival curves for 12‐month survival, for Charlson, MIS, Barthel and Fried. Scale classifications: Charlson: Cutoff 10.5 pts. MIS: cutoff 7.5 pts. Barthel: cutoff 72.5 pts. Fried: cutoff 2.5 pts. Statistical significance: *p* < 0.05.

Figure 2 presents the Kaplan–Meier survival curves for 12‐month survival, between the different scales used in relation to the normality marker cutoff points described in Figure 1. As we can see, all four variables identify patients who die earlier within the one‐year period.

**FIGURE 3:**
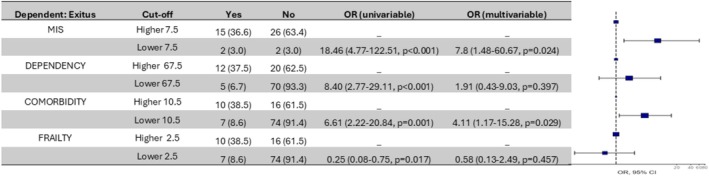
Risk of death according to the cutoff points for MIS, dependency, comorbidity and frailty scales, with corresponding odds ratios (OR) and confidence intervals (CI) shown.

Finally, Figure 3 graphically represents the risk of death according to the cutoff points that mark mortality. As seen in the univariable analysis (Figure 3A), we find a significant risk of death for all four scales, which is lost in the multivariable analysis (Figure 3B) for the dependence and frailty scales. The risk of death is significantly higher only with the established cutoff point in the comorbidity and malnutrition scales. Similarly, Table S1, which presents hazard ratios from Cox proportional hazards models, reinforces the Figure 3 finding. Although the univariable analysis using odds ratios showed significant associations for all four scales (comorbidity, malnutrition, dependence and frailty), only the malnutrition scales retained their predictive power in the multivariable models as aetiology and haemodialysis dose and adequacy are taken into account.

## 4 Discussion

Our study defines characteristics that determine mortality in haemodialysis patients over 75 years old, establishing cutoff points for commonly used scales of comorbidity, malnutrition, dependence and frailty. The risk of death for patients reaching these cutoff points is notably higher, particularly for comorbidity (Charlson scale) and malnutrition (MIS scale).

The high percentage of our elderly haemodialysis population in moderate to severe dysfunction stages aligns with previous studies [25, 26]. Our findings show 59% of patients presenting high comorbidity (Charlson scale), 55% moderately to extremely malnourished (MIS scale), 21% dependent and 49% frail, corroborating the complex health status of this population.

The cutoff point of 9.5 on the Charlson scale for predicting mortality (AUC 0.788) is higher than previously reported cutoffs for the general population [9] and aligns with studies on haemodialysis patients [17, 27]. This higher cutoff likely reflects the added comorbidity burden in elderly dialysis patients.

For the MIS scale, our cutoff of 7.5 points (AUC 0.844) corresponding to very severe malnutrition is consistent with other studies in dialysis populations [18, 28]. The strong association between malnutrition and mortality in our study underscores the critical importance of nutritional status in this population, as highlighted by other researchers [29, 30].

The Barthel scale cutoff of 67.5 points (AUC 0.79) for dependency aligns with previous studies showing the impact of functional status on outcomes in elderly dialysis patients [31, 32]. Our findings on the association between inability to walk, institutionalization and mortality further emphasize the importance of maintaining functional status.

The FRIED frailty scale cutoff of 2.5 points (AUC 0.719) is lower than the traditional cutoff of 3 for frailty [12]. This suggests that in elderly haemodialysis patients, even prefrail status may significantly impact mortality risk, a finding supported by other studies in CKD populations [33].

Our multivariable analysis highlighting comorbidity and malnutrition as the strongest predictors of mortality aligns with previous research emphasizing these factors in dialysis outcomes [34]. This underscores the need for comprehensive assessment and management of comorbidities and nutritional status in elderly dialysis patients.

In addition to comorbidity and malnutrition, cardiovascular disease plays a crucial role in the mortality of dialysis patients. Cardiovascular complications are the leading cause of death in this population, often driven by the interplay of hypertension, left ventricular hypertrophy, diabetes, cholesterol and chronic inflammation [35]. In particular, the presence of ischaemic heart disease, heart failure and arrhythmias has been associated with increased mortality in elderly haemodialysis patients [36]. Identifying and managing cardiovascular risk factors, including better control of blood pressure, volume status and lipid metabolism, may be essential for improving survival.

Infections represent another significant contributor to mortality in dialysis patients. This population is highly susceptible to infections due to immune system dysfunction, frequent vascular access manipulation and hospitalization exposure. Bacteraemia and respiratory infections are among the most common causes of infection‐related death in haemodialysis patients [37]. Hospitalizations for infections have been linked to poor prognosis and functional decline in haemodialysis patients, with factors such as advanced age, cognitive impairment and immobility contributing to this functional decline [38]. Strategies to reduce infection rates, such as improved vascular access management, vaccination programmes and early detection of infectious complications, should be a priority in dialysis care.

Our study has defined characteristics that determine mortality in haemodialysis patients over 75 years old, establishing cutoff points for commonly used scales of comorbidity, malnutrition, dependence and frailty. The risk of death for patients reaching these cutoff points is significantly higher. Specifically, on the Charlson comorbidity scale, the risk of death is 6.2 times higher, and on the MIS nutrition scale, it is 5.7 times higher. These findings underscore comorbidity and malnutrition as the main determinants of mortality in our patients.

Classifying our elderly haemodialysis population with the four scales, we found a high percentage in the moderate to severe dysfunction stages. Specifically, 63 (59%) of the patients presented high comorbidity according to the Charlson scale (using the median as the cutoff point, which coincides with the more than 8 points described in the literature) [17]. Regarding the MIS scale, 59 (55%) were moderately to extremely malnourished, 22 (21%) were dependent and 49 (49%) were frail patients. We found no differences between sexes.

The best point to discriminate mortality determined by the ROC curves was 9.5 points for the Charlson scale, with a sensitivity of 65% and specificity of 81%, AUC 0.788 (95% CI: 0.65–0.88). This corresponds to high comorbidity as described in the literature for both the normal population and haemodialysis patients, in which a significant increase in 1‐year mortality is described in those with scores higher than 8 points [17]. For the malnutrition‐inflammation scale, the cutoff point determining mortality was 7.5 points, with a sensitivity of 71% and specificity of 86%, AUC 0.844 (95% CI: 0.73–0.93), which corresponds to a state of very severe malnutrition (above 7 points) on the MIS scale [18].

For the Barthel dependency scale, the statistically calculated cutoff point was 67.5 points, with a sensitivity of 71% and specificity of 69%, AUC 0.79 (95% CI: 0.68–0.79), corresponding to moderate dependence in the use of the scale [19]. The Barthel test measures the individual's capabilities in performing activities of daily living, which are significantly impacted by associated comorbidity and malnutrition in dialysis patients. Regarding frailty measured by FRIED, the cutoff point determining mortality was 2.5 points, with a sensitivity of 63% and specificity of 75%, AUC 0.719 (95% CI: 0.56–0.83), corresponding to the pre‐frail state [12], although close to the frail state, which would be three points.

Finally, the results shown highlight the importance of comorbidity and malnutrition as determining factors of mortality. In the univariable analysis, all scales significantly increase the risk of death when patients reach the established cutoff points. However, in the multivariable analysis, only comorbidity and malnutrition maintain their significant difference with more than 6.20 (1.64–26.37, *p* = 0.009) times the risk of death for patients who exceed the cutoff point in Charlson and 5.65 (1.13–43.01, *p* = 0.052) times more risk for patients who exceed the cutoff point in MIS. It is important to note that although overall comorbidity was quantified using a global score, we did not assess individual conditions (e.g., diabetes, cardiovascular disease and cancer) in a qualitative manner. This limitation may have affected our ability to fully capture the influence of these specific factors on mortality. In addition to comorbidity and malnutrition, cardiovascular disease and infections play a crucial role in the mortality of HD patients. For instance, ischemic heart disease and heart failure have been associated with higher mortality, whereas infections—due to compromised immunity and vascular access issues—further contribute to adverse outcomes.

Our findings indicate that although dependence and frailty appear significant in univariable models, only comorbidity and malnutrition retain their predictive power after adjustment when etiological factors and measures of dialysis adequacy are integrated into the analysis. This suggests that inadequate dialysis dosing—manifested by lower Kt/V and reduced HD hours—coupled with a higher MIS may synergistically increase mortality risk. Therefore, optimizing dialysis delivery and enhancing nutritional support could be pivotal strategies in improving outcomes for elderly haemodialysis patients.

Several limitations of our study should be noted. First, the single‐centre design and relatively small sample size may limit generalizability. Multicentre studies with larger cohorts are needed to confirm our findings. Second, the observational nature of the study precludes causal inferences. Third, we did not account for changes in patient status over time, which could influence outcomes. Longitudinal studies with repeated measures could provide more dynamic insights. Additionally, we did not explore the impact of specific comorbidities or the potential interactions between different risk factors. Due to limitations in the medical records, the specific causes of death could not be accurately ascertained, and only total mortality was recorded. More granular analysis of these aspects could provide further insights into mortality risk. Although we intended to analyse causes of death, limitations in medical records prevented clear identification in many cases, allowing us to report only total mortality. Finally, our study did not include measures of quality of life or patient‐reported outcomes, which are increasingly recognized as important in geriatric nephrology [39].

Prospective studies examining the impact of interventions targeting malnutrition and comorbidity management on mortality in elderly dialysis patients are needed. The potential benefits of exercise programmes or nutritional supplementation in improving functional status and reducing mortality risk should be explored [40]. Additionally, given the significant role of cardiovascular disease and infections in this population, future research should focus on optimizing cardiovascular prevention strategies and infection control measures to improve survival. Multidisciplinary approaches integrating nephrology, cardiology and infectious disease specialists may provide more comprehensive management tailored to the needs of elderly dialysis patients. Further research is also needed to understand the complex interplay between frailty, malnutrition and comorbidity in this population. Developing integrated risk assessment tools that combine these factors could enhance prognostication and guide individualized care plans. Lastly, studies incorporating patient preferences and goals of care are crucial. As the dialysis population ages, aligning treatment approaches with patient values and quality of life considerations becomes increasingly important [41].

In conclusion, our study highlights the critical role of comorbidity and malnutrition in determining mortality risk among elderly haemodialysis patients. The identified cutoff points for various assessment scales offer practical tools for risk stratification. Future research and clinical practice should focus on developing and implementing targeted interventions to address these key risk factors and improve outcomes in this vulnerable population.

## Ethics Statement

The current study was approved by the ethics committee of the Hospital Universitario Fundación Jiménez Díaz (Act No. 03/19) and complied with the standards recognized by the Declaration of Helsinki of the World Medical Association, as well as the Standards of Good Clinical Practice, in addition to compliance with Spanish legislation on biomedical research (Law 14/2007).

## Consent

Informed consent was obtained from all subjects involved in the study.

## Conflicts of Interest

The authors declare no conflicts of interest.

## Supporting information


**Table S1:**Cox proportional hazard models for different etiologies, comorbidities, and clinical parameters in patients with CKD. Univariable and multivariable hazard ratios (HR) with 95% confidence intervals (CI) and *p*‐values are shown. The reference groups for categorical variables are indicated by "—".

## Data Availability

The database employed for the current study is available upon request to the corresponding authors.
